# Evaluation of Acute Terminal Ileitis in Hospitalized Patients: Development of a Predictive Model to Distinguish Crohn’s Disease from Other Etiologies

**DOI:** 10.3390/jcm13175030

**Published:** 2024-08-25

**Authors:** Anton Bermont, Naim Abu-Freha, Refael Aminov, Sergei Vosko, Haim Shirin, Daniel L. Cohen

**Affiliations:** 1 The Gonczarowski Family Institute of Gastroenterology and Liver Diseases, Shamir (Assaf Harofeh) Medical Center, Zerifin 70300, Israel; sergeivosko@gmail.com (S.V.); haimsh@shamir.gov.il (H.S.); danielc@shamir.gov.il (D.L.C.); 2 Institute of Gastroenterology and Hepatology, Soroka University Medical Center, Faculty of Health Sciences, Ben-Gurion University of the Negev, Beer-Sheva 84105, Israel; abufreha@yahoo.de; 3 Internal Medicine Department, Shamir (Assaf Harofeh) Medical Center, Zerifin 70300, Israel; refael.aminovd@gmail.com

**Keywords:** Crohn’s disease, terminal ileitis, abdominal pain

## Abstract

**Background/Objectives**: Terminal ileitis (TI) is often identified on CT scans in emergency settings. Diagnosing Crohn’s disease (CD) as a cause of TI is crucial due to its significant long-term implications. This study aimed to differentiate CD from other causes of acute TI and develop a predictive model for CD diagnosis. **Methods**: A retrospective case-control study was conducted at Shamir Medical Center including adults diagnosed with acute TI from January 2012 to December 2020. Patients with a history of inflammatory bowel disease or prior intestinal surgery were excluded. Patients were categorized into CD and non-CD groups based on their subsequent clinical course. A logistic regression model was developed and subsequently validated with additional patients hospitalized between 2021 and 2023. **Results**: Among 135 patients, 37 (27.4%) were diagnosed with CD. CD patients were younger (median age 27 vs. 39 years, *p* = 0.003), predominantly male (83.8% vs. 51%, *p* = 0.001), and had higher rates of chronic abdominal pain, diarrhea, anemia, and weight loss prior to hospitalization. Significant laboratory differences included higher platelet counts (*p* = 0.006) and lower mean corpuscular volume (MCV) (*p* = 0.001) in CD patients. Radiologic signs of complicated disease were more common in CD (35.1% vs. 4.1%, *p* < 0.001). The predictive model incorporating gender, abdominal pain history, and MCV showed an area under the curve (AUC) of 0.87, with a sensitivity of 100% and specificity of 63.6% in the validation group of 18 patients. **Conclusions**: This study identified key predictors of CD in patients presenting with acute TI and developed a predictive model with a substantial diagnostic capability. Use of this model for early identification and treatment of CD may potentially improve patient outcomes. Further prospective validation of this model is warranted.

## 1. Introduction

Terminal ileitis (TI), inflammation within the terminal ileum, is often identified on CT scans in the emergency-room setting. The differential diagnosis of TI is vast and encompasses a spectrum of etiologies ranging from reactive changes secondary to appendicitis, infections, drug reactions, neoplastic processes, vasculitis, and Crohn’s disease (CD) [1,2]. In regions such as Israel, where the incidence of CD is notably high at 14.9 per 100,000 individuals, it is important to identify cases of CD who present with TI [3].

Symptoms such as abdominal pain, weight loss, and chronic diarrhea often raise the suspicion of CD, particularly in younger patients [4]. However, these symptoms do not consistently correlate with the diagnosis [5,6]. Establishing the diagnosis of CD requires a combination of endoscopy and histological examination [7] Historically, long-term follow-up was deemed necessary to definitively distinguish those with CD, a chronic disease, from other patients presenting with an acute resolving form of TI from infectious etiologies [8]. More recent efforts have aimed to integrate commonly available laboratory results and imaging studies with initial clinical symptoms to develop risk-stratification tools and models. These tools are designed to predict the likelihood of small bowel CD at the first patient presentation, although accurately diagnosing new-onset CD remains a challenge [9].

Emerging evidence suggests that the course of autoimmune conditions, such as rheumatoid arthritis (RA), may be altered favorably by the early initiation of anti-inflammatory therapy [10,11]. Similarly, early intervention in CD may alter the disease’s trajectory, potentially preventing progression to irreversible bowel damage and the need for surgery [12]. Indeed, the transition to irreversible bowel damage in CD can occur within the first year of disease onset [13]. In one population-based cohort study, 18.6% of patients with Crohn’s disease experienced penetrating or stricturing complications within 90 days after diagnosis [14]. The consequences of a delayed diagnosis can be profound, often necessitating urgent and early surgical intervention for CD-related complications [15]. Conversely, initiating early treatment with agents such as thiopurines or TNF inhibitors within the first year of diagnosis is associated with reduced rates of surgical intervention and improved efficacy during maintenance therapy in adults [16,17,18].

This study aimed to investigate the causes and outcomes of patients presenting with acute TI, as diagnosed on a CT scan, particularly focusing on its role as a marker of the initial presentation of CD. By comparing those subsequently diagnosed with CD and those with other etiologies, we sought to construct and validate a statistical model to accurately predict which patients with TI would present with new-onset CD.

## 2. Materials and Methods

### 2.1. Patients

The study included adults aged 18 and older diagnosed with TI based on a CT scan showing inflammation of the terminal ileum. TI cases associated with abscesses, fistulae, or colitis were included. All abdominal CT scans were performed according to the standard protocol for emergency room studies and interpreted by a senior radiologist. Patients whose TI resulted from conditions such as appendicitis or diverticulitis, those with a known history of inflammatory bowel disease (IBD), or those who had previous intestinal surgery were excluded.

### 2.2. Ethical Considerations

The study was approved by the medical center’s Ethics Committee (IRB No: 064-23-ASF; approval date 16 April 2023). The need for informed consent was waived given the anonymous and retrospective nature of the study.

### 2.3. Study Outcomes

The primary objective of the study was to identify predictors for acute TI secondary to CD as opposed to other causes of TI. The secondary objective was to determine the outcomes of patients hospitalized with acute TI secondary to new-onset CD.

### 2.4. Study Designs

A single-center, retrospective case-control study was performed to evaluate patients diagnosed with acute TI and hospitalized at Shamir Medical Center between January 2012 and December 2020. Data were systematically collected from the patients’ medical records, focusing on demographic information (age, gender), past medical history, clinical presentation (details of symptoms at presentation and chronic symptoms during the one year prior to hospitalization), laboratory test results (complete blood count [CBC]; biochemical profiles; C-reactive protein [CRP] levels; ALT; AST; neutrophil-to-lymphocyte ratio [NLR]; platelet-to-lymphocyte ratio [PLR]), imaging studies (associated findings on CT scans relevant to the TI diagnosis), and endoscopic examinations.

### 2.5. Follow-Up

Patients were followed longitudinally to collect data on their health outcomes following hospitalization, with particular attention to subsequent diagnoses and required surgical and medical interventions. 

Patients were divided into two comparison groups: one consisting of patients subsequently diagnosed with Crohn’s disease following their initial TI presentation (CD group) and another comprising patients with TI not attributed to Crohn’s disease based on diagnostic investigations and follow-up (non-CD group). The CD was diagnosed according to ECCO recommendation based on colonoscopy results, with biopsy results from TI in association with the clinical course and CT findings over follow-up [19]. By comparing the CD group and non-CD group, a statistical model was developed for predicting CD at the time of first presentation with TI (see below). To validate the model, an additional 18 patients with TI hospitalized between May 2022 and July 2023 were analyzed to test whether the model could accurately predict cases of CD. The patients included in the validation cohort met the same inclusion and exclusion criteria as the initial TI cohort.

### 2.6. Statistical Analyses

Categorical variables were summarized as frequency and percentage. Continuous variables were evaluated for normal distribution using a histogram. Since all continuous variables were skewed, they were reported as median and interquartile range. The Chi-square test and Fisher Exact Test were applied to compare categorical variables between those with and without CD, while the Mann–Whitney Test was used to compare continuous variables.

Multivariable logistic regression using a forward likelihood ratio selection method was used to identify predictors for CD and to build the prediction model (*p* < 0.05 was set for variable inclusion). The area under the receiver operating characteristic curve, the discrimination slope, and the box plot were used to evaluate how the model could allow discrimination between patients with and without CD. The discrimination slope was calculated as the absolute difference in the average predictions for patients with and without CD. The Maximal Youden index was used to identify the cut-off value. Sensitivity and specificity in the learning and validation groups were reported.

All statistical tests were 2-sided and *p* < 0.05 was considered statistically significant. SPSS was used for all statistical analyses (IBM SPSS Statistics for Windows, version 28, IBM Corporation, Armonk, New York, NY, USA, 2021).

## 3. Results

### 3.1. Demographic Data and Clinical Presentation

**Figure 1:**
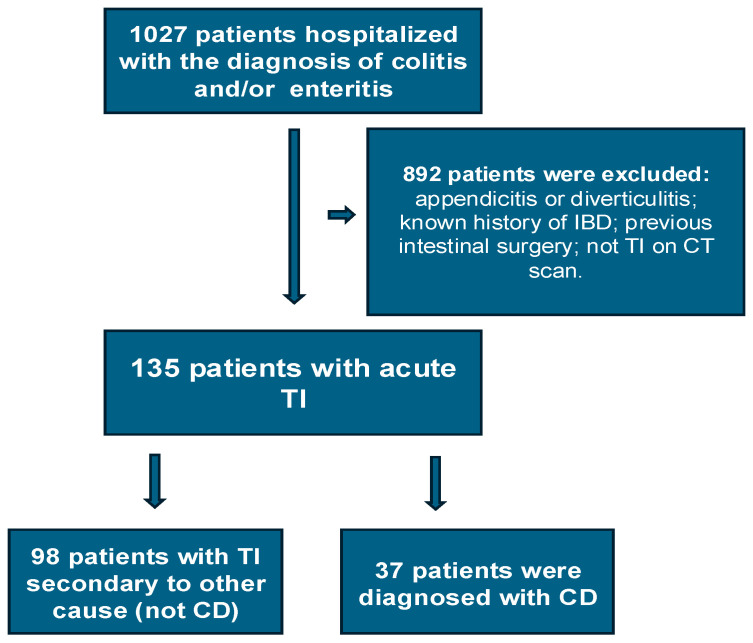
Flowchart of the study population.

In total, 1027 patients were hospitalized with enteritis or colitis during the study period. Of these, a total of 135 patients met the inclusion criteria and became the TI cohort (Figure 1). Of these, 98 (72.6%) were in the non-CD group and 37 (27.4%), in the CD group. Details of their demographics and medical history can be seen in Table 1. The median age for the entire cohort was 35 years, with the non-CD group being older (median age 39 years) compared to the CD group (median age 27 years) (*p* = 0.003).

Of the total cohort, 81 (60%) were males and 54 (40%) were females. Males constituted a higher percentage in the CD group compared to the non-CD group (83.8% vs. 51%, *p* = 0.001).

A positive family history of CD was more prevalent in the CD group (12.1%) compared to the non-CD group (2.3%), which was statistically significant (*p* = 0.049). 

Patients in the CD group experienced a significantly higher incidence of complaints of abdominal pain within the year prior to hospitalization (45.9% vs. 13.3%, *p* < 0.001) compared to the non-CD group, as well as a significantly higher rate of diarrhea (21.6% vs. 8.2%, *p* = 0.04). Additionally, patients with CD had a higher incidence of anemia (21.6% vs. 8.2%, *p* = 0.041) and weight loss (27.3% vs. 4.7%, *p* = 0.001) prior to hospitalization. 

Overall, 58 (43%) patients reported smoking, with no significant difference between the groups (39.8% in non-CD vs. 51.4% in CD, *p* = 0.226).

Nearly all patients presented acute abdominal pain (98.5%), with no significant difference between groups. Other symptoms, such as peritoneal signs, diarrhea, and fever, were similar between the groups (Table 2).

### 3.2. Laboratory Test Results

In a comparative study of laboratory results at the time of hospitalization between the CD group and the non-CD group, several significant differences were observed (Table 3). Platelet counts were significantly higher in the CD group (mean 273 × 10^9^/L vs. 224 × 10^9^/L in non-CD, *p* = 0.006). The mean corpuscular volume (MCV) was lower in the CD group (mean 82 fL vs. 87 fL in non-CD, *p* = 0.001). Additionally, the neutrophil-to-lymphocyte ratio (NLR) and platelet-to-lymphocyte ratio (PLR) were higher in the non-CD group (NLR 6.1 and PLR 181 vs. NLR 4.5 and PLR 141 in CD, *p* values 0.011 and 0.001, respectively).

### 3.3. CT Scan Results in CD vs. Non-CD PATIENTS

On CT, a significantly higher percentage of patients with CD (35.1% vs. 4.1% in non-CD, *p* < 0.001) presented radiologic signs of complicated disease (including collections, fistulas, or obstruction). Colitis was notably less common in CD patients, with only one case (2.7%) (Table 4).

### 3.4. Evaluation and Treatment during Hospitalization

Fecal cultures were obtained from only 25 patients (18.5%). Of these, the cultures were positive for *Campylobacter jejuni* in only two cases. Most patients (96.3%) were treated with a regimen of empiric antibiotics (either Ceftriaxone with Metronidazole or Ciprofloxacin with Metronidazole). Only two patients received steroids. All patients improved with empiric treatment and were discharged home.

### 3.5. Follow-Up and Outcomes

Follow-up data were available for a mean of 5.7 ± 2.5 years. Fourteen patients (37.8%) in the CD group were readmitted to the hospital within half a year since their first hospitalization. There were no readmissions in the non-CD group. Ten patients (27%), all from the CD group, underwent surgery (ileocecectomy) over the next few years due to CD, with seven of them undergoing surgery within a year of their TI presentation. No other specific causes from TI in the non-CD group were found during follow-up.

### 3.6. Multivariable Analysis and Model Construction

A logistic regression analysis was performed using the forward selection method, which considered all the significant variables in the univariate analyses to identify factors that increase the risk of CD in patients presenting with TI (Table 5). This revealed that being male (odds ratio [OR] = 6.25, 95% CI: 2.12–18.46), having a history of chronic abdominal pain prior to TI presentation (OR = 4.21, 95% CI: 1.44–12.34), and having a lower MCV (OR = 0.87, 95% CI: 0.80–0.95) all significantly increased the risk of having CD.

Hence, the probability of having CD could be calculated by using the following equation:

P(CD) = 1/(1 + exp(−Z)).

Z = 8.926 + 1.833 if male + 1.439 if pain before − 0.135 × MCV

where P(CD)—probability of having CD; MCV—mean corpuscular volume.

**Figure 2:**
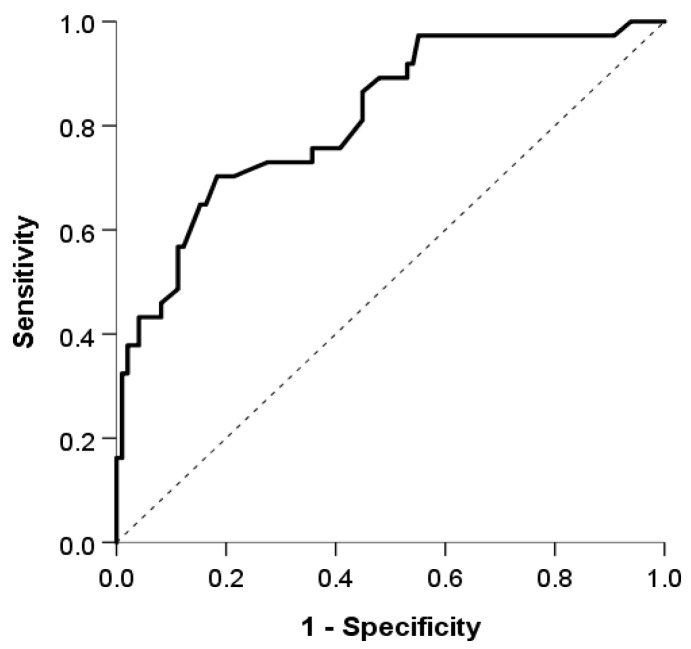
The area under the the receiver operating characteristic curve used to evaluate the discrimination ability of our model.

**Figure 3:**
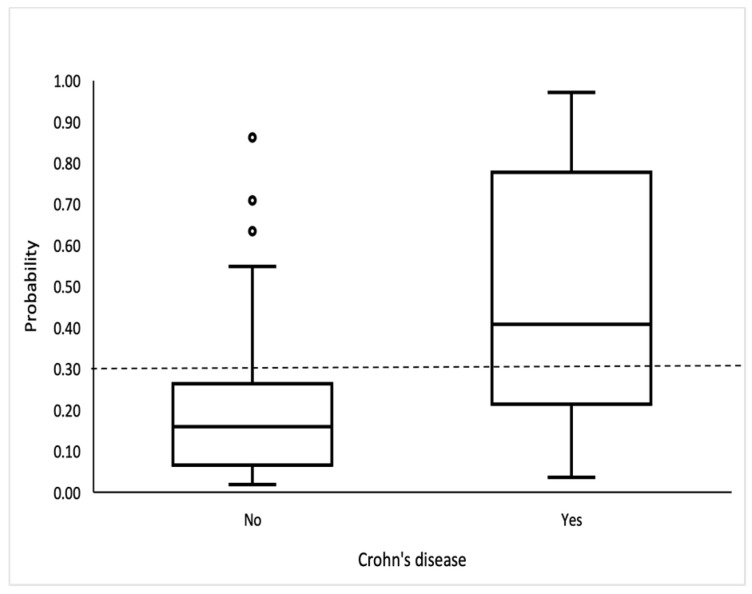
Box-and-whisker plot shows probability for CD. Bottom and top of boxes indicate 25th and 75th percentiles, respectively. Horizontal lines inside boxes indicate median values.

The logistic model showed good discrimination and calibration abilities. The area under the curve was 0.815 (95% CI: 0.734, 0.897) (Figure 2), and the discrimination slope was 0.30. The Maximal Youden index indicated a cutoff value of 0.3, which provided a sensitivity of 70.3%, specificity of 81.6%, positive predictive value (PPV) of 59.1%, and negative predictive value (NPV) of 87.9%. (Figure 3).

### 3.7. Validation of the CD Prediction Model

Eighteen patients with acute TI were included in a validation group. With a mean follow-up of 18.3 ± 5.7 months, seven (38.9%) were subsequently diagnosed with CD. Overall, 55.6% were men and the median age was 28 years (21–54), with the non-CD age being 29 (21–62) and the CD age, 27 (21–51).

The model showed a good discrimination ability between patients with and without CD with an AUC of 0.87 (95% CI 0.704–1.00, *p* < 0.01), using the cut-off value of 0.3. The sensitivity was 100%, specificity was 63.6%, NPV was 100%, and PPV was 63.6%.

## 4. Discussion

Our study provided descriptions and outcomes of patients presenting with acute TI which allowed us to create and validate a model aimed at identifying those presenting with new-onset CD. CD is notoriously challenging to manage, largely due to its complex and often unpredictable clinical course. Among the 37 patients with CD included in our study, a significant proportion (37.8%) required hospital readmission within six months of their initial diagnosis. Furthermore, 27% of these patients underwent surgical interventions, most of which occurred within a year since their initial hospitalization. The results are consistent with earlier research showing a high rate of surgery within the first 3 years after being diagnosed with CD, especially within the first 6 months [20]. However, the surgical rate was significantly higher than previously reported data, which indicated a 16.3% risk of surgery one year after CD diagnosis [18].

Nearly all patients with acute TI (96.3%) received empirical antibiotic treatment, with all showing positive clinical and laboratory responses, and were discharged for outpatient follow-up. Only 18.5% completed investigations including fecal cultures, with *Campylobacter jejuni* identified in just two instances. Although a specific cause of inflammation was not identified in most cases, the rapid and favorable response to antibiotics suggests that infectious causes were likely the primary etiology. However, this approach may not be applicable in regions where tuberculosis (TB) is endemic, as TB-associated ileitis can mimic the clinical and radiologic presentation of CD [21,22].

Patients with CD as the cause of TI had a more complicated course with higher rates of readmission and surgery compared to the milder outcomes of the non-CD patients. This finding aligns with the broader literature on CD which characterizes the disease as a chronic, relapsing condition with a high risk of complications and poor long-term outcomes if not diagnosed and managed promptly [13,14].

Demographic analysis revealed that patients with TI due to CD were younger and predominantly male. These findings align with previous research indicating a higher incidence of CD in younger individuals [3,23]. However, while male predominance was more significant in patients hospitalized with TI due to CD, this differs from epidemiologic studies where no significant difference between males and females with CD was observed [3,24]. Additionally, a positive family history of similar conditions was significantly more common in the CD group, which is consistent with findings from previous studies [24,25].

Patients with TI due to CD experienced more frequent gastrointestinal symptoms prior to their acute TI episode, including abdominal pain, diarrhea, anemia, and weight loss, compared to their non-CD counterparts. In the multivariable analysis, the most significant of these was a history of abdominal pain, which may reflect the chronic inflammatory nature of small bowel CD [26,27].

Laboratory results were comparable between patients with CD and those without. Key inflammatory markers, including WBC count, neutrophil count, and CRP levels, were elevated to similar extents in both groups, indicating that these markers are not effective in identifying the underlying cause of TI in acute situations. Notably, patients with CD had somewhat higher platelet counts, possibly pointing to a distinct element of their inflammatory response. This contrasts with prior research where both WBCs and platelet levels were markedly higher in CD patients and used as part of a predictive model for TI in primary care settings [9]. The non-CD group exhibited an elevated neutrophil-to-lymphocyte ratio (NLR) and platelet-to-lymphocyte ratio (PLR), suggesting a different inflammatory profile. Although hemoglobin levels were similar across groups, the MCV was significantly lower in the CD group, which may indicate chronic iron deficiency due to the illness.

Radiologic findings revealed that CD patients were more likely to present with complications such as collections, fistulas, or obstructions, reflecting the intramural inflammation in CD. In contrast, the presence of colitis was markedly higher in non-CD patients, likely due to infectious enterocolitis, and this too may assist clinicians in differentiating CD from other causes of ileitis.

Due to the diagnostic complexities of CD, several studies have focused on developing predictive models to aid physicians in identifying patients likely to have CD [28,29,30]. A notable advancement in this field came from Sachdeva et al., who integrated clinical, laboratory, radiological, and colonoscopic data into a robust algorithm. This algorithm adeptly classifies patients with chronic isolated TI into specific and nonspecific etiologies, demonstrating excellent diagnostic accuracy [31]. In addition, Shen and colleagues have created a predictive model targeting small bowel CD, specifically for evaluating lower abdominal symptoms in a primary care setting [9]. This model incorporates both clinical assessments and laboratory data, including inflammatory markers. However, the efficacy of these models was reduced in cases of acute TI, where inflammatory markers are elevated across various conditions and endoscopy findings are less discriminating.

Therefore, to address this gap, our study introduces a tailored model for identifying CD in patients with acute TI. Utilizing forward stepwise logistic regression, we identified gender, complaints of abdominal pain prior to the acute TI episode, and MCV as significant predictors of CD. With an AUC of 0.87, sensitivity of 70.3%, specificity of 81.6%, PPV of 59.1% and NPV of 87.9%, our model demonstrates a substantial capability to differentiate between CD and non-CD cases. This model, when used in conjunction with clinical judgment and radiologic findings, could significantly enhance the early diagnosis of CD, leading to timely and more targeted therapeutic interventions.

While this study provides valuable insights, there are some limitations due to its retrospective nature and single-center design. There was the potential for selection bias in identifying cases of TI, and potential issues with the accuracy of medical records and lack of prospectively collected data on symptoms. Future research should focus on multi-center, prospective studies with larger study populations to validate these findings and refine the predictive model in a larger group of validation patients. Incorporating more specific data, such as genetic markers, medication use, physical activity, and detailed dietary histories could also enhance the model’s accuracy. Moreover, examining the impact of early diagnostic interventions on long-term outcomes of CD could substantiate the benefits of early diagnosis and treatment initiation.

## 5. Conclusions

This study identified predictors of new-onset CD amongst patients presenting with acute TI through a comprehensive assessment of clinical, laboratory, and imaging characteristics. The predictive model developed provides a valuable framework for the early identification of CD, which is crucial for improving patient outcomes through timely and targeted therapeutic strategies. Future prospective studies further validating this predictive model may aid healthcare providers in assessing patients with TI, ultimately improving the quality of life and disease prognosis for CD patients.

## Figures and Tables

**Table 1:** Demographics and symptoms before hospitalization.

	All *n* = 135 (%)	Non-CD *n* = 98 (%)	CD *n* = 37 (%)	*p* Value
Age (median [IQR])	35 (24;52)	39 (27;54)	27 (22;38)	0.003
Gender				
Male	81 (60)	50 (51)	31 (83.8)	0.001
Female	54 (40)	48 (49)	6 (16.2)	
Smoking	58 (43)	39 (39.8)	19 (51.4)	0.226
Family history of CD (n = 119)	6 (5)	2 (2.3)	4 (12.1)	0.049
Abdominal pain within the past year	30 (22.2)	13(13.3)	17 (45.9)	<0.001
Diarrhea within the past year	16 (11.9)	8 (8.2)	8 (21.6)	0.04
Anemia within the past year (n = 134)	16 (11.9)	8 (8.2)	8 (21.6)	0.041
Weight loss (n = 119)	13 (9.6)	4 (4.7)	9 (27.3)	0.001

**Table 2:** Clinical presentation in hospitalization.

Clinical Presentation	All *n* = 135 (%)	Non-CD *n* = 98 (%)	CD *n* = 37 (%)	*p* Value
Abdominal pain	133 (98.5)	96 (98)	37 (100)	>0.999
Peritoneal signs	17 (12.6)	13 (13.3)	4 (10.8)	>0.999
Diarrhea	63 (46.7)	49 (50)	14 (37.8)	0.206
Fever	53 (39.3)	39 (39.8)	14 (37.8)	0.835

**Table 3:** Laboratory test results from the first day of hospitalization.

	All *n* = 135 (IQR)	Non-CD *n* = 98 (IQR)	CD *n* = 37 (IQR)	*p* Value
WBCs (×10³/µL)	10.9 (8.2;13.9)	10.4 (7.5;13.5)	11.7 (10.2;14.1)	0.116
HB (g/dL)	13.7 (12.8;14.9)	13.7 (12.8;15.0)	13.7 (12.7;14.3)	0.374
PLTs (×10³/µL)	232 (187;280)	224 (187;261)	273 (190;346)	0.006
MCV (fL)	86 (82;89)	87 (84;90)	82 (77;85)	0.001
LYMs (×10³/µL)	1.5 (1.2;2.0)	1.6 (1.2;2)	1.5 (1.1;1.8)	0.063
NEUs (×10³/µL)	8.3 (5.6;11.2)	7.7 (5.0;11.1)	9.3 (7.1;11.4)	0.073
CRP (mg/L)	62 (27;127.7)	58 (18;122)	86 (40;134)	0.098
ALT (U/L)	14 (10;19)	15 (11;21)	12 (7.5;17.0)	0.008
AST (U/L)	16 (13;21)	17 (14;22)	15 (10.5;18.0)	0.004
NLR		4.5 (2.9;8.6)	6.1 (4.7;9.3)	0.011
PLR		141 (104;184)	181 (137;301)	0.001

Values are presented as median (interquartile range). WBCs: White blood cells; NEUs: neutrophils; LYMs: lymphocytes; PLTs: platelets; ALT: alanine aminotransferase; AST: aspartate aminotransferase; NLR: neutrophil-to-lymphocyte ratio; PLR: platelet-to-lymphocyte ratio.

**Table 4:** CT findings in patients with TI.

CT Findings	CD *n* = 37 (%)	Non-CD *n* = 98 (%)	*p* Value
TI with collection, fistula, or obstruction	13 (35.1)	4 (4.1)	<0.001
TI with colitis	1 (2.7)	14 (14.3)	0.068
TI alone	23 (62.2)	80 (81.6)	0.018

**Table 5:** Multivariate analysis of risk factors for CD in patients presenting with TI.

Variable	OR	95% CI	*p* Value
Gender (Male)	6.25	2.12–18.46	0.001
Pain before	4.21	1.44–12.34	0.009
MCV (fL)	0.87	0.80–0.95	0.002

## Data Availability

The research data are not publicly available due to privacy or ethical consideration. Once this manuscript is accepted for publication, delinked data without personal privacy could be provided upon reasonable request.
